# HERALD (Health Economics using Routine Anonymised Linked Data)

**DOI:** 10.1186/1472-6947-12-24

**Published:** 2012-03-29

**Authors:** Muhammad J Husain, Sinead Brophy, Steven Macey, Leila M Pinder, Mark D Atkinson, Roxanne Cooksey, Ceri J Phillips, Stefan Siebert

**Affiliations:** 1 Keele Management School, Keele University, Staffordshire, ST5 5BG, UK; 2 College of Medicine, Swansea University, Wales, SA2 8PP, UK; 3 College of Human and Health Sciences, Swansea University, Wales SA2 8PP, UK

## Abstract

**Background:**

Health economic analysis traditionally relies on patient derived questionnaire data, routine datasets, and outcomes data from experimental randomised control trials and other clinical studies, which are generally used as stand-alone datasets. Herein, we outline the potential implications of linking these datasets to give one single joined up data-resource for health economic analysis.

**Method:**

The linkage of individual level data from questionnaires with routinely-captured health care data allows the entire patient journey to be mapped both retrospectively and prospectively. We illustrate this with examples from an Ankylosing Spondylitis (AS) cohort by linking patient reported study dataset with the routinely collected general practitioner (GP) data, inpatient (IP) and outpatient (OP) datasets, and Accident and Emergency department data in Wales. The linked data system allows: (1) retrospective and prospective tracking of patient pathways through multiple healthcare facilities; (2) validation and clarification of patient-reported recall data, complementing the questionnaire/routine data information; (3) obtaining objective measure of the costs of chronic conditions for a longer time horizon, and during the pre-diagnosis period; (4) assessment of health service usage, referral histories, prescribed drugs and co-morbidities; and (5) profiling and stratification of patients relating to disease manifestation, lifestyles, co-morbidities, and associated costs.

**Results:**

Using the GP data system we tracked about 183 AS patients retrospectively and prospectively from the date of questionnaire completion to gather the following information: (a) number of GP events; (b) presence of a GP 'drug' read codes; and (c) the presence of a GP 'diagnostic' read codes. We tracked 236 and 296 AS patients through the OP and IP data systems respectively to count the number of OP visits; and IP admissions and duration. The results are presented under several patient stratification schemes based on disease severity, functions, age, sex, and the onset of disease symptoms.

**Conclusion:**

The linked data system offers unique opportunities for enhanced longitudinal health economic analysis not possible through the use of traditional isolated datasets. Additionally, this data linkage provides important information to improve diagnostic and referral pathways, and thus helps maximise clinical efficiency and efficiency in the use of resources.

## Background

Health service research have tended to rely on data generated from randomised controlled trials (RCT), observational studies based on patient derived questionnaire data, and routinely assembled data abstracted from the primary and secondary care patient record - popularly known as routine data [1]. Data generated from these three routes are predominantly used as stand-alone datasets, and alongside non-health data such as demographic and geographical data [1,2]. The health sector analyses are enriched when the patient-level data generated from these different sources are linked. Many countries worldwide already routinely capture health care data that can be used for such purposes. For example, the Scottish Morbidity Linked Dataset which encompasses Scottish Health Survey records, linked to NHS acute and psychiatric hospital records, cancer, and death registrations provides powerful research database http://www.esds.ac.uk/government/shes/; in France, record linkage between a hospital database and the French national mortality database offers new prospects for large prognostic studies based on hospital data [3]; and in Norway, the Medical Birth Registry of Norway is routinely linked with the Central Population Register, and can be linked with the other central health registers. This paper discusses the potential methodological advantages in the conduct of health economics analyses using patient-derived questionnaire data linked with routinely collected information and secondary care clinical datasets available in Wales, United Kingdom, with examples from a research cohort.

## Methods

### SAIL databank

In order to realise the potential of electronically-held routinely collected information to conduct and support health-related research, the Health Information Research Unit (HIRU) at the College of Medicine at Swansea University, as part of the Welsh Assembly Government's commitment to the UK Clinical Research Collaboration (UKCRC), has set up the Secure Anonymised Information Linkage (SAIL) databank [4,5]. The SAIL databank brings together and links a wide range of person-based data. SAIL utilises a split-file approach to anonymisation to overcome issues of confidentiality and disclosure in health-related data warehousing by creating personal-level unique and encrypted identifiers for merging information from various sources [4,5]. The range of complementary sets of data includes clinical data from rheumatologists, existing routinely collected datasets such as the General Practice (GP) records, outpatient (OP) clinical data, inpatient (IP) episodes, accident and emergency (A&E) department, pathology data, NHS administrative register, breast and cervical cancer screening data, all Wales injury surveillance system, all Wales perinatal survey, congenital anomaly register and information service, birth and death data from the Office for National Statistics, and social services databases.

### Data linkage

HIRU uses the MACRAL (Matching Algorithm for Consistent Results in Anonymised Linkage) algorithm to create encrypted Anonymised Linking Field (ALF) for each individual [4]. The ALFs are mainly created based on the patient's NHS number; and if the NHS number is absent in a dataset, a mixture of other identifying variables like forename, surname, gender, postcode of residence, and date of birth are used for probabilistic matching, while maintaining complete anonymity for the end users [4]. This linkage allows us to follow the patient pathway through the NHS system both retrospectively and prospectively from a reference date (e.g. questionnaire completion date). This system also allows linkage of data collected through patient questionnaires with other routinely collected datasets in the SAIL system.

### Data linkage with PAS cohort

As part of the Medical Research Council (MRC) Patient Research Cohort Initiative, a cohort of people with ankylosing spondylitis (AS), i.e. the Welsh population-based ankylosing spondylitis (PAS) cohort, has been developed using data collected from patient completed questionnaires linked with routine data [6]. The study aims to recruit 1000 AS patients living in Wales and currently about 500+ AS patients are participating. This study has ethical approval from the London Multi-centre Research Ethics committee and the written consent of participants was obtained according to the Declaration of Helsinki. For the PAS cohort, the data collected from patients with a diagnosis of AS can be linked to other routinely collected datasets using the SAIL system. To highlight the potential benefits of using the linked routine data, this paper uses information on healthcare visits as reported by the AS patients through questionnaires and explores patient pathways in terms of actual events obtained from the linked GP, OP, IP, and A&E datasets in the SAIL databank.

### Potential benefits of using linked data

The paper attempts to demonstrate the strength of data linkage in extracting complementary information from routine sources that are beyond the scope and time horizon of the study data, and does not intend to test hypotheses with regards to the data quality pertaining to the specific variable of interest or the individual datasets. The potential benefits of using linked data are discussed below.

### 1. Retrospective and prospective tracking of patient pathways

With data spanning multiple years and the ability to link records across several datasets, it is possible using SAIL to track the healthcare utilisation history of patients in receipt of some form of intervention for a given condition across multiple healthcare sectors both before and after their index/reference healthcare event. Therefore, the SAIL data linkage system allows tracking of the patient pathways, both retrospectively and prospectively. Linkage with GP data system provides information about patients' primary care events going back many years including; previous diagnoses, referrals, presenting symptoms, investigation results and previous medications. This dataset can also be used to follow the patient at every visit to the GP and therefore record the development of associated conditions and use of co-medications. Linkage with IP data will record all hospital visits, surgery and hospital treatment. Linkage with the mortality datasets will ensure the dataset remains relevant and can examine survival of included patients. Linkage with A&E datasets will give information on emergency visits.

### 2. Validation of patient-reported recall data

The use of linked routine data allows cross-checking of patient-reported recall data with actual health care events at the personal level. The inherent limitations (or strengths) of the data quality pertaining to the survey questionnaires under the recall method can be flagged; and an assessment of the generalisability of the patient-reported data can be made. On the other hand, data obtained from routinely collected data systems often require careful interpretation with respect to their quality, validity, timelines, bias, confounding and statistical stability [1]. With the triangulation of datasets in the SAIL system, the validity and reliability of single datasets can be assessed [6]. The triangulation process will at least flag the discrepancies, and we can then have an idea about any quality issue pertaining to both the routine and questionnaire data. However, this paper does not intend to make assertions with regards to the data quality of individual datasets, and views the linking of datasets primarily as a source of extracting complementary information which are beyond the scope and time horizon of traditional study data.

### 3. Objective measure of the cost of illness

Cost of illness studies are typically subject to a degree of scrutiny with regards to the sources and methods of estimating the quantities and prices, the specification of study perspective, and the identification of the timeframe to which the costs apply [7-9]. The use of linked data enhances the precision of the healthcare use information and the timelines within which the costs incurred; and therefore will help provide an objective estimate of the cost and burden of diseases to the funders, health service (NHS in the United Kingdom), society and the individual at each stage of disease over a prolonged period of time.

In many conditions there is a delay between the onset of symptoms and establishing a diagnosis, during which period the patients still utilise healthcare resources. The linked routine data can provide information about the patients' visits to health care facilities during this symptomatic pre-diagnosis period, when the requirement for diagnostic investigations is often greatest. For example, within the SAIL data system, using the encrypted ALF, we can identify patients from a cohort of any particular disease who were diagnosed during a reference time (as indicated by first appearance of a specific diagnostic read code); and link those with various datasets (e.g. GP data, IP hospital admissions, OP, A&E data etc.) to track their pre-diagnosis visits to healthcare facilities since the date the symptom onset (as reported by the patients or established from the GP or A&E records). This allows one to compare the extent of health service utilisation, and therefore related costs, before and after the symptoms developed. In addition, one can also calculate the costs as a result of delayed diagnosis.

The linked healthcare analysis within SAIL need not be confined to deducing the extent of health service utilisation during the pre- and post-diagnosis illness periods but can additionally be performed to ascertain the size of the direct medical costs associated with the index illness that are incurred across different healthcare sectors. This is possible, for example, with the combined use of the cost figures included in the Trust Financial Return 2 (TFR2) accounts [10] that incorporate expenditures relating specifically to A&E attendances, IP admissions and OP contacts; and the cross-sector (i.e. primary care, secondary care, IP, OP, A&E etc.) health services utilisation at the individual level obtained from the linked data system. A system such as the SAIL system therefore not only allows the index event for a given condition to be identified but additionally introduces a longitudinal, temporal, dimension to the analysis as each of the healthcare sectors captured within SAIL can be searched for multiple years pre- and post-index healthcare event to determine the extent of health service utilisation and direct medical costs, made possible by the inclusion of an ALF within all of the SAIL datasets. This provides a more objective estimate of the actual costs of chronic conditions and any interventions.

### 4. Healthcare pathways and referral history

A retrospective analysis of the patient's healthcare history can identify the types of referrals to healthcare services made at different points in time, thus, giving an assessment of health service usage and recommendations for improving patient care pathways. In particular, the linked GP data would provide important information to improve diagnostic and referral pathways, and thus maximise clinical efficiency and efficiency in the use of resources. The temporal aspects of the linked data sets also help conducting event history analysis, survival analysis and other relevant statistical and econometric models.

### 5. Profiling of patients

The linked SAIL data includes diverse sets of information, which enables profiling and stratification of patients relating to disease manifestations and severity, lifestyle, co-morbidities, and associated costs. Additionally, given that many chronic conditions have heterogeneous manifestations with a variable course and unpredictable episodes of exacerbation, the analysis can be carried out under several person stratification schemes based on severity of disease, various demographic attributes, and socio-economic conditions. This stratification will facilitate early targeting of interventions to patients at highest risk, thereby improving the cost-effectiveness ratio of these interventions.

## Results

### An example using a patient with ankylosing spondylitis

Here we present an example of one AS patient's health service usage history by tracking the healthcare events through the linked datasets and comparing this with self-reported data. To preserve complete anonymity, the actual dates are modified by replacing with fictitious dates. As part of the PAS cohort study, the patient completed a questionnaire during the first week of November 2009, in which s/he was asked to recall the number of visits to the GP, OP, IP, A&E, and to various health professionals during the three months before the questionnaire completion date. The patient reported 4 GP visits, 1 OP visit, 1 IP visit, no A&E visit, and visited a rheumatologist, a radiologist, and a chiropractor once each. Distances to the healthcare facilities were 1 mile, 3.5 miles, and 3 miles for GP, OP, and IP, respectively. In each case the patient used their own car; and was accompanied by someone during the GP and IP visits. The patient also reported having taken pain reducing medicines (paracetamol, ibuprofen, and naproxen); having undergone an MRI scan and had blood and urine tests during the 3 months recall period.

**Figure 1:**
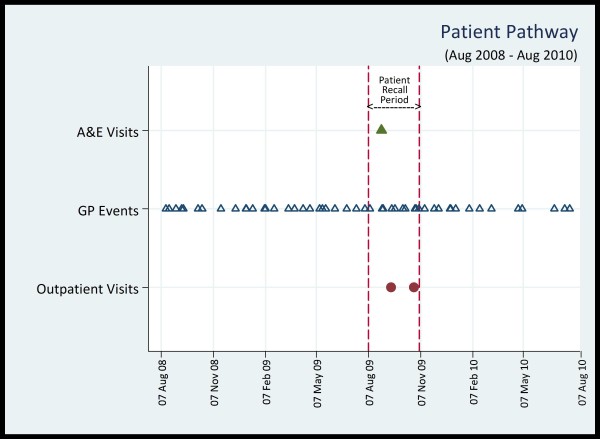
**Healthcare Pathway of an AS Patient**. Each shape indicates a separate event (date) captured in the relevant datasets. The X-axis captures events for a period of two years (i.e. 730 days). Therefore, the GP event markers appear almost overlapped when events occurred in two consecutive dates (e.g. 31^st ^August and 1^st ^September). The questionnaire completion date is 05 November, 2009. The three month patient recall period is captured by the two vertical dashed lines, indicating the dates 07 August and 05 November. There are 10 GP events during this time interval, including the overlapped ones.

Using the unique ALF, we tracked the patient's healthcare pathways through the routine data in SAIL system. Figure 1 plots the patient's healthcare events from the OP, GP, and A&E datasets for a 2 year period (i.e. August 2008 to August 2010), which represents the timeline approximately one year before and one year after the completion date of the questionnaire by the patient.

The linked routine data show 10 GP events, 2 OP visits and 1 A&E visit during the 3 month recall period. There is no IP visit recorded during this period. Therefore, the self-reported IP visit in the questionnaire may actually be an A&E visit, which would correlate with the routine data. Out of those 10 GP events, not all of them are physical visits by the patients, but may include any event (e.g. letter encounter, prescription collection, telephone conversation etc.). Further exploration of the GP read codes and descriptions for these 10 GP events yields information about medication, tests, and other GP related encounters, as shown in Table 1. Retrospective and prospective tracking of events reveals that there are 51 such GP events during the 2 year period (Figure 1). There are no OP, IP, or A&E visits before or after the recall period, indicating the danger of extrapolating patient reported 3 month recall data in the questionnaire over a longer period (e.g. one year). However, going further back through the linked data system in terms of the timeline (not shown in the figure), it was found that the patient made 4 OP visits during August, October, and November of 2005; and 1 IP visit on the first week of June 1999.

**Table 1:** Patient's GP Read codes during the 3 month recall period

GP Event Date	(sl) GP Read Codes: Descriptions	Comment
10 August	(i) fh1k: PREMIQUE 0.625 mg/5 mg tablets; (ii) b211: BEMDROFLUAZIDE 2.5 mg tablets; (iii) b211: BENDROFLUMETHIAZIDE 2.5 mg tablets; (iv) bxd2: SIMVASTATIN 20 mg tablets; (v) Bd35: ATENONOL 50 mg tablets	GP visit /Prescription collection

31 August	1969: Abdominal pain	System reporting of A&E admission on 30 August

01 September	Discharge Summary	System reporting of A&E discharge summary (ref: 30 August A&E admission)

17 September	(i) fh1k: PREMIQUE 0.625 mg/5 mg tablets; (ii) b211: BEMDROFLUAZIDE 2.5 mg tablets; (iii) b211: BENDROFLUMETHIAZIDE 2.5 mg tablets; (iv) bxd2: SIMVASTATIN 20 mg tablets; (v) Bd35: ATENONOL 50 mg tablets; (vi) j2ck: NAPROXEN 250 mg e/c tablets; (vii) 246..: O/E blood pressure; (viii) di21: PARACETAMOL 500 mg; (ix) 8B4: Repeat Prescription.	GP Visit

23 September	(i) 52...: Plain Radiography; (ii) 52....:Plain X-Ray	GP visit

07 October	(i) bd35: ATENONOL 50 mg tablets; (ii) b211: BEMDROFLUAZIDE 2.5 mg tablets; (iii) bxd2: SIMVASTATIN 20 mg tablets; (iv) 8CB..: Had a chinwag with patient; (v) 8CB..: Had a discussion with patient.	GP visit

11 October	9 N4..: Failed encounter	No visit

28 October	(i) b211: BEMDROFLUAZIDE 2.5 mg tablets; (ii) b211: BENDROFLUMETHIAZIDE 2.5 mg tablets; (iii) bxd2: SIMVASTATIN 20 mg tablets; (iv) Bd35: ATENONOL 50 mg tablets (v) j2ck: NAPROXEN 250 mg e/c tablets	GP visit/prescription collection

30 October	(i) j2ck: NAPROXEN 25 mg e/c tablets	Prescription collection

04 November	(i) 9 N36: Letter from specialist; (ii) Letter from consultant	No visit

### Examples using the PAS cohort linked to GP data system

Using the GP data we tracked about 183 AS patients from the PAS cohort retrospectively and prospectively from the date of questionnaire completion to gather the following information: (a) number of GP events; (b) presence of a GP parent family 'drugs' read codes; and (c) the presence of a GP parent family 'diagnostic' read codes.

#### GP events and visits from the linked routine and questionnaire data

Table 2 presents the average number of GP events for the AS patients grouped under several stratification schemes based on baseline disease severity score (low and high BASDAI); disease function score (low and high BASFI); age, sex, and the age of the onset of first symptoms. Results are presented for the retrospective 3 months recall period, 1 year period, and 5 years period - the questionnaire completion dates being the reference date for each patient. In the last column of Table 2, we present the self-reported number of GP visits during the 3 month period from the questionnaires. As mentioned earlier the GP 'event' and 'visits' are to be construed differently. The GP event is defined as the unique dates for each patient where we can find GP read codes indicating administrative actions, referrals, visits, telephone conversation, prescription collection, symptoms, diagnosis, prescription drugs etc., pertaining to the particular patient. The GP visits refer to the physical visits to the GP by the patient. In the questionnaire the patients were asked about the visits to the GP. In the presence of numerous read codes, it is beyond the scope this paper to identify 'visits' from within the 'events'. Nevertheless, the number of actual visits obtained from the questionnaire can be used as complementary information as to what proportion of the GP events were GP visits.

**Table 2:** Number of GP 'events' from linked GP data and GP 'visits' from questionnaire data

	Number of GP 'events' from linked GP data (Mean [95% CI])			Number of GP 'visits' from Questionnaire data
**Patient Stratification Schemes**	**5 year period**	**1 year period**	**3 month recall** **period**	**3 months recall period**

**Disease Severity Index**				

Low (BASDAI < 40) (n = 72)	**61.92** [47.86-75.64]	**12.92** [9.96-15.87]	**2.81** [2.06-3.55]	**1.31** [0.98-1.63]

High (BASDAI > = 40) (n = 72)	**80.28** [64.32-96.23]	**17.79** [14.04-21.54]	**4.25** [3.22-5.28]	**1.78** [1.34-2.21]

*Ratio (high/low BASDAI)*	***1.30***	***1.38***	***1.51***	***1.36***

**Disease Functional Index**				

Low (BASFI < 40) (n = 71)	**49.15** [36.99-61.32]	**11.20** [8.38-14.02]	**2.75** [1.97-3.52]	**1.21** [0.87-1.55]

High (BASFI > = 40) (n = 73)	**92.27** [76.34-108.21]	**19.40** [15.73-23.07]	**4.29** [3.29-5.29]	**1.86** [1.45-2.28]

*Ratio (high/low BASFI)*	***1.88***	***1.73***	***1.56***	***1.54***

**Age**				

Age < 50 (n = 68)	**45.96** [34.05-57.87]	**10.46** [7.54-13.38]	**2.53** [1.65-3.41]	**1.44** [1.00-1.89]

Age > = 50 (n = 76)	**93.43** [77.95-108.92]	**19.74** [16.27-23.21]	**4.42** [3.53-5.31]	**1.63** [1.30-1.97]

*Ratio (Age > = 50/Age < 40)*	***2.03***	***1.89***	***1.75***	***1.13***

**Sex**				

Male (n = 113)	**75.52** [63.43-87.61]	**16.07** [13.32-18.83]	**3.69** [2.95-4.43]	**1.50** [1.21-1.79]

Female (n = 31)	**54.58** [32.82-76.34]	**12.74** [7.82-17.66]	**2.93** [1.61-4.26]	**1.68** [0.95-2.40]

*Ratio (Male/Female)*	***0.72***	***0.79***	***0.79***	***1.12***

**Disease Symptom**				

First Symptom at age < = 30 (n = 112)	**66.03** [54.94-77.11]	**14.39** [11.86-16.93]	**3.29** [2.61-3.98]	**1.46** [1.17-1.76]

First Symptom at age > 30 (n = 32)	**88.47** [60.53-116.40]	**18.72** [12.45-24.99]	**4.34** [2.68-6.01]	**1.81** [1.11-2.51]

*Ratio (first sym. age > 30/age < = 30)*	***1.34***	***1.30***	***1.32***	***1.24***

Table 2 shows that the patients with low disease severity have less GP visits and events than the patients with high disease severity. The patients with low disease severity, had 2.81, 12.92, and 61.92 events recorded in the GP system during the 3 months, 1 year, and 5 year retrospective period as opposed to 4.25, 17.79, and 80.28 events for the high disease severity groups. These ratios are consistent with those for the number of self-reported visits obtained from the questionnaires during the 3 month recall period, which are 1.31 visits for the low severity group and 1.78 for the high severity group. The 3 month GP events and self-reported GP visits in the high disease severity group were 1.51 and 1.36 times respectively more than in the low disease severity group and the between-group relative differences are largely consistent throughout the 5 year period (see Table 2). The same strategy can be applied to the other stratification models. The data in Table 2 indicates that the largest discrepancies between self-reported GP visits and routine data GP events are in the groups stratified by age and gender. We postulate that this suggests that older patients with AS (age ≥50) either tend to under-report GP visits or have more non-visit related GP events (e.g. for prescriptions) compared to younger patients, or that the reverse is true for younger patients. Similar hypotheses can be generated for female and male patients. This may have important implications as AS affects men more commonly than women, with onset in late teens or early adult years.

#### Linked routine GP data for medications

Table 3 indicates the presence of parent drugs in the AS patients' GP history. The GP read codes starting with small letters *a-z *indicate the parent drug family the prescribed medicines belong to. Starting with the small letters *a-z*, the drug related GP read codes extends up to 4 more sub-digits to specifically identify the drug. It is beyond the scope of this paper to go beyond the first parent groups. Table 3 shows, in different retrospective time spans, how many patients' GP read codes include the mentioning of a particular drug code at least once. It is evident that the highest number of patients is prescribed musculoskeletal and joint drugs (read code '*j*'), followed by the central nervous system drugs (which include analgesics) *(d)*. Other drug classes frequently recorded in the AS patients include gastro-intestinal system drugs *(a)*, cardiovascular system drugs *(b)*, and the skin drugs (m), which is consistent with the association of these diseases with AS. One could go beyond the parent read codes and specifically identify exactly which drug was prescribed at which date. The drug codes for the 3 months after (prospective) the dates of baseline questionnaire completion for 103 AS patients are shown in the last column of Table 3.

**Table 3:** Presence of Parent Drug & Appliances Read Codes for the AS Patients

Parent Read Code	Disease Group	Retrospective				Prospective
		
		10 year (n = 183)	5 year (n = 164)	1 year (n = 147)	3 months (n = 120)	3 months (n = 103)
**a**	Gastro-intestinal system drugs	123	103	75	47	42

**b**	Cardiovascular system drugs	69	65	50	39	36

**c**	Respiratory system drugs	74	56	30	18	20

**d**	Central nervous system drugs	147	125	83	52	40

**e**	Drugs for infectious diseases	139	114	59	20	18

**f**	Endocrine drugs	63	56	29	19	13

**g**	Obs/gynae/urinary drugs	42	33	22	8	6

**h**	Malignant & immunosuppr. drug	19	13	9	8	8

**i**	Haematology/dietetic drugs	54	44	29	18	17

**j**	Musculoskeletal & joint drugs	157	134	89	59	52

**k**	Eye drugs	61	48	26	11	9

**l**	Ear, nose & oropharynx drugs	61	48	16	7	6

**m**	Skin drugs	105	90	46	23	17

**n**	Immunology drugs & vaccines	48	38	15	3	0

**o**	Anaesthetics	10	7	1	0	0

**p**	Appliances & reagents etc.	41	37	14	8	8

**q**	Incontinence Appliances	2	2	2	1	1

**s**	Contrast media	8	8	5	3	2

Note: Baseline questionnaire completion is the reference date. The last column applies to the patients whose data in the GP system exist at least 3 months post questionnaire completion date

#### Routine GP data for associated diagnosis

Table 4 similarly shows the presence of parent disease diagnostic read codes (start with capital letters *A-Z*) in the GP data system for the AS patients at 10 years, 5 years, 1 year and 3 months retrospective, and 3 months prospective time span, relative to the date of questionnaire completion. The most frequent disease group as indicated by the read codes fall under the musculoskeletal/connective tissue *(N)*, skin/subcutaneous tissue disease *(M)*, nervous system/sense organ diseases *(F)*, respiratory system disorders *(H)*, digestive system disorders *(J)*, symptoms, signs, ill-defined conditions *(R)*, and infectious/parasitic diseases *(A)*. These are consistent with the conditions that are associated with AS (e.g. psoriasis, uveitis, colitis) or complicate the treatment (e.g. respiratory infections in patients on immunosuppressive therapy). Again, further exploration of the parent read codes by going beyond the first digit would reveal the specific disease diagnosis.

**Table 4:** Presence of Parent Diagnosis Read Codes for the AS Patients

Parent Read Code	Disease Group	Retrospective				Prospective
		
		10 year (n = 183)	5 year (n = 164)	1 year (n = 147)	3 months (n = 120)	3 months (n = 103)
**A**	Infectious/parasitic diseases	49	35	15	5	1

**B**	Neoplasms	26	16	1	0	0

**C**	Endocr/nutr/metabolic/immun.	42	26	7	0	0

**D**	Blood/blood forming organs	14	9	2	1	1

**E**	Mental disorders	35	26	10	4	0

**F**	Nervous system/sense organ	92	78	25	13	3

**G**	Circulatory system diseases	67	44	6	3	4

**H**	Respiratory system disorders	88	72	25	4	5

**J**	Digestive system disorders	81	47	14	6	7

**K**	Genitourinary system diseases	47	36	8	1	0

**L**	Pregnancy/childbirth/puerperium	1	0	0	0	0

**M**	Skin/subcutaneous tissue diseases	94	77	27	3	6

**N**	Musculoskeletal/connective tissue	149	110	36	9	11

**P**	Congenital anomalies	0	0	0	0	0

**Q**	Perinatal conditions	0	0	0	0	0

**R**	Symptoms, signs, ill-defined conditions	60	48	14	4	4

**S**	Injury and self poisoning	56	31	9	1	2

**T**	Causes of injury/poisoning	21	12	3	0	0

**U**	External causes morbdty/mortlty	2	1	0	0	0

**Z**	Unspecified conditions	29	22	5	3	1

Note: Baseline questionnaire completion is the reference date. The last column applies to the patients whose data in the GP system exist at least 3 months post questionnaire completion date

### Examples using the PAS cohort linked to OP and IP data systems

We tracked 236 and 296 AS patients retrospectively through the OP and IP data systems respectively to derive the average number of OP visits and IP admissions made by the patients grouped under different stratification schemes. The results are presented in Table 5. The table has two panels - columns 1-4 relate to OP visits, and columns 5-8 relate to the IP admissions.

**Table 5:** Number of outpatient (OP) visits and inpatient (IP) admissions from linked and questionnaire data

	Number of OP Visits from linked data (Mean [95% CI])			# of self-reported OP visits from Questionnaire	Average Number of IP admissions from linked data (Mean [95% CI])			# of self-reported IP admissions from Questionnaire
	**1**	**2**	**3**	**4**	**5**	**6**	**7**	**8**

**Patient Stratification Schemes**	**5 year**	**1 year**	**3 month recall period**	**3 months recall period**	**5 year**	**1 year**	**3 month recall period**	**3 months recall period**

**Disease Severity Index**								

Low (BASDAI < 40) (n = 119 & 133)	**16.78** [13.50-20.06]	**3.84** [2.96-4.72]	**0.87** [0.64-1.09]	**1.06** [0.76-1.36]	**2.43** [1.38-3.48]	**0.59** [0.26-0.91]	**0.11** [0.05-0.17]	**0.08** [0.03-0.13]

High (BASDAI > = 40) (n = 117 & 163)	**21.89** [16.87-26.91]	**6.12** [4.29-7.95]	**1.51** [1.06-1.97]	**2.55** [1.54-3.55]	**3.01** [2.27-3.76]	**0.74** [0.51-0.97]	**0.17** [0.10-0.23]	**0.14** [0.07-0.21]

**Disease Functional Index**								

Low (BASFI < 40) (n = 110 & 117)	**14.17** [11.23-17.12]	**3.05** [2.28-3.81]	**0.73** [0.53-0.93]	**0.90** [0.62-1.18]	**2.31** [1.06-3.55]	**0.46** [0.12-0.80]	**0.11** [0.05-0.17]	**0.05** [0.01-0.09]

High (BASFI > = 40) (n = 126 & 179)	**23.80** [18.93-28.67]	**6.65** [4.92-8.39]	**1.59** [1.16-2.02]	**2.58** [1.64-3.52]	**3.04** [2.40-3.69]	**0.81** [0.58-1.04]	**0.16** [0.10-0.22]	**0.16** [0.09-0.23]

**Age**								

Age < 50 (n = 90 & 164)	**18.29** [14.64-21.94]	**5.07** [3.10-7.03]	**1.30** [0.81-1.79]	**1.72** [0.80-2.65]	**1.91** [1.29-2.54]	**0.49** [0.24-0.74]	**0.16** [0.08-0.25]	**0.05** [0.01-0.09]

Age > = 50 (n = 146 & 192)	**19.95** [15.65-24.24]	**4.91** [3.79-6.03]	**1.12** [0.84-1.39]	**1.84** [1.20-2.48]	**3.21** [2.31-4.11]	**0.77** [0.51-1.04]	**0.13** [0.08-0.18]	**0.15** [0.08-0.22]

**Sex**								

Male (n = 183 & 218)	**18.20** [14.85-21.55]	**4.35** [3.48-5.22]	**1.02** [0.79-1.25]	**1.60** [1.08-2.12]	**2.47** [1.96-2.97]	**0.55** [0.40-0.69]	**0.13** [0.08-0.18]	**0.10** [0.05-0.15]

Female (n = 53 & 76)	**23.15** [16.50-29.80]	**7.11** [3.71-10.52]	**1.77** [0.98-2.57]	**2.47** [0.95-4.00]	**3.59** [1.62-5.57]	**1.03** [0.40-1.66]	**0.16** [0.06-0.26]	**0.14** [0.05-0.24]

**Disease Symptom**								

First Symptom at age < = 30 (n = 182 & 226)	**19.04** [15.51-22.57]	**4.81** [3.59-6.04]	**1.23** [0.92-1.54]	**1.85** [1.20-2.51]	**2.38** [1.95-2.81]	**0.58** [0.42-0.73]	**0.14** [0.09-0.19]	**0.10** [0.05-0.15]

First Symptom at age > 30 (n = 54 & 70)	**20.22** [14.69-25.75]	**5.50** [3.86-7.14]	**1.04** [0.68-1.40]	**1.61** [0.95-2.27]	**3.97** [1.71-6.23]	**0.99** [0.33-1.65]	**0.16** [0.05-0.26]	**0.17** [0.06-0.28]

#### OP visits

**Figure 2:**
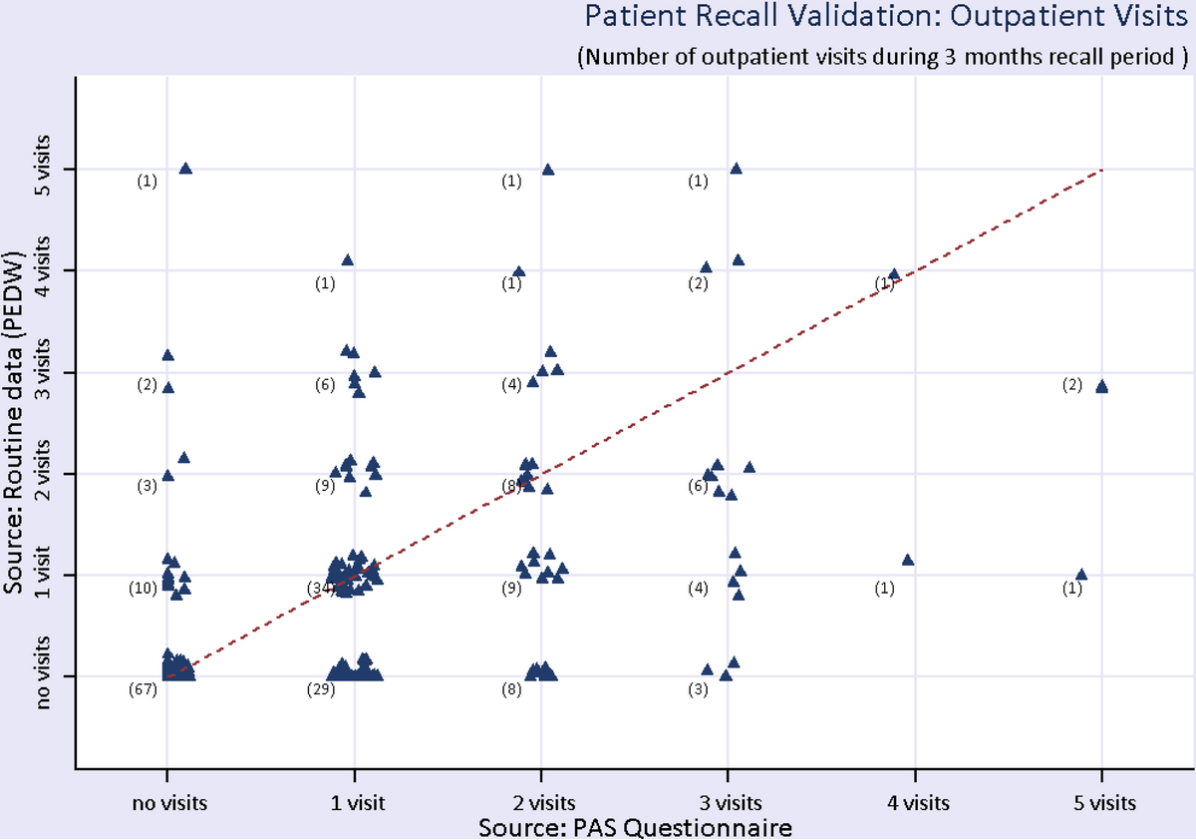
**Outpatient (OP) visits from routine data and self-reported patient recall**. Brackets indicate numbers of patients.

The estimates in columns 3 and 4 of Table 5 report the average number of visits for the 3 months recall period obtained from the routine data system and the questionnaires respectively. In principle, the numbers in these two columns should match as they relate to OP visits only. It is observed that patients in all groups tend to overestimate the number of OP visits in the questionnaire. The over-reporting of OP visits is most marked in those with high disease activity (BASDAI) and functional impairment (BASFI). We postulate that one reason for this may be as a result of these patients finding it more difficult to physically attend OP clinics due to their greater disease activity and disability. Again, this may have important implications when using patient-reported data to estimate utilisation of healthcare resources. Figure 2 shows the number of self-reported and recorded OP visits by the AS patients. The x-axis shows the number of visits during the 3 months recall period self-reported in the questionnaire and the y-axis shows the corresponding number of visits obtained from the records of OP data. The numbers in the bracket is the number of patients. For instance, out of 79 patients who reported in the questionnaire to have visited once to the OP during the 3 months recall period, we found 29 patients with zero visit, 34 patients with 1 visit, 9 patients with 2 visits, and 7 patients more than 2 visits. It can be seen that in general, the patients overestimated the number of OP visits when completing the questionnaires. 29 patients reported an OP visit that was not recorded during the 3 month recall period, highlighting issues with using recall data.

#### IP visits

In the second panel of Table 5 (i.e. columns 5-8), we tracked 296 AS patients through the IP data system to obtain the number of IP admissions. Again, in principle, columns 7 and 8 should match, and indeed these results for IP admissions are more similar than the corresponding data for OP visits (columns 3 and 4). The results in columns 7 and 8 suggest that, in contrast to OP visits, patients tend to underestimate the number of IP visits. Younger patients and those with less severe disease severity (BASDAI) and functional impairment (BASFI) were most likely to underestimate IP admissions. We postulate that this is because the admission in these patients was more likely to be for a reason unrelated to their AS, and therefore overlooked and not reported in a questionnaire for an AS study.

Tracking through the retrospective data of those who had at least one IP admission, Table 6 indicates additional complementary information on the number of days spent in hospital. This information was not captured in the questionnaire data. These data indicate that older patients, those with longer disease duration, higher disease activity and functional impairment spent significantly more days in hospital than their relevant comparison groups. This may also have been a contributory factor to the relative under-reporting of IP admissions in patients with lower disease severity or functional impairment.

**Table 6:** Inpatient (IP) Spells: Average Number of days as IP from Linked IP data

Patient Stratification Schemes	Average Number of days as IP from Linked IP data (Mean [# attended][95% CI])		
	
	5 years	1 year	3 months recall period
**Disease Severity Index**			

Low (BASDAI < 40)	**10.82** [n = 100] [7.48-14.16]	**5.06** [n = 36] [3.04-7.07]	**1.43** [n = 14] [0.94-1.92]

High (BASDAI > = 40)	**12.97** [118] [9.16-16.79]	**5.62** [58] [3.87-7.37]	**2.33** [24] [1.02-3.64]

**Disease Functional Index**			

Low (BASFI < 40)	**9.31** [81] [6.03-12.59]	**4.04** [25] [1.51-6.57]	**1.25** [12] [0.96-1.54]

High (BASFI > = 40)	**13.57** [137] [9.99-17.15]	**5.90** [69] [4.36-7.44]	**2.35** [26] [1.13-3.56]

**Age**			

Age < 50	**7.11** [75] [4.74-9.48]	**3.37** [30] [1.75-4.98]	**1.4** [15] [0.94-1.86]

Age > = 50	**14.55** [143] [10.90-18.19]	**6.36** [64] [4.63-8.09]	**2.39** [23] [1.03-3.75]

**Sex**			

Male	**12.13** [158] [9.22-15.04]	**4.93** [69] [3.47-6.38]	**2.00** [27] [0.90-3.10]

Female	**11.72** [58] [6.14-17.31]	**6.83** [24] [3.79-9.87]	**2.00** [10] [0.57-3.43]

**Disease Symptom**			

First Symptom at age < = 30	**10.96** [170] [8.14-13.77]	**4.65** [75] [3.31-6.00]	**1.38** [29] [1.12-1.64]

First Symptom at age > 30	**15.63** [48] [9.59-21.66]	**8.37** [19] [4.76-11.98]	**4.00** [9] [0.37-7.63]

## Discussion

The above examples demonstrate that linked routine data enables validation and clarification of patient reported data; the retrospective and prospective tracking of the patient healthcare utilization and pathways; and the referral history in a cohort of patients with AS. Such analysis makes it possible to deduce whether the anonymised individuals in question were suffering any common co-morbidities, in receipt of healthcare treatment prior to the occurrence of the reference event, whilst it also allows any frequent complications requiring medical attention in the days, months, years following the event (which could be an intervention or questionnaire) to be identified. From the methodological perspective, any linkage system would add new dimensions and perspective to traditional health related research (e.g. complement and enhance the results of RCTs), as a resource for clinical audits, and in a variety of health impact assessment exercises. For example, the longitudinal routine data would allow an assessment of the impact of specific healthcare interventions on subsequent healthcare utilisation (e.g. A&E visits or hospital admission). Important limitations of solely relying on questionnaire data include reliance on accurate patient recall and that the healthcare events of interest may not occur within the limited recall period (e.g. 3 months), but just before the recall period or after the completion date of the questionnaire. This makes extrapolation of the questionnaire data for an extended period of time unreliable. The longitudinal linked routine data comes into aid in this respect.

The linkage of the questionnaire data from the PAS patients with the GP data as shown above enhances and helps make sense of the rich information obtained from the GP Read codes. This constitutes a rich health history for these AS patients, for whom we can carry out patient pathway analysis from various clinical and economic aspects. In particular, in keeping with other AS cohorts, these patients had an average lag of about 8 years from symptom onset to AS diagnosis, on which we can conduct pre- and post-diagnosis analyses of health care utilisation.

Again, a matrix of traits based on the PAS questionnaire information linked with the SAIL data system will help profiling of AS patients for health and other related interventions. Table 7 summarises the types of information gathered through the PAS questionnaires, which could all be linked with the routine data sources as well as various demographic, socio-economic, and environmental attributes of the patients.

**Table 7:** PAS Questionnaire Contents

Questionnaire (Time Interval)	Summary of Information
Baseline (0)	Co-morbidities, family history, age of diagnosis and first symptoms, disease activity [11], function [12], Quality of life (EQ-5D) [13], and visits to health professionals

Not at work (3 Months)	Previous occupation, activity impairment questionnaire

At work (3 months)	Work questionnaire, including information about current and previous occupation, activity impairment and work limitations questionnaire (WLQ) [14], work productivity and activity impairment questionnaire (WPAI-SHP) [15,16]

AS costs (9 months)	AS Cost questionnaire including detailed patient-level information about visits to health care facilities, professionals, AS related pathology and other tests, other conditions, medications, costs of various aspects of treatment and disability

Exercise and Fatigue (15 months)	International Physical Activity Questionnaire (IPAQ) [17], disease activity, function, Behavioural Regulation in Exercise Questionnaire (BREQ-2) [18], Pittsburgh Sleep Quality Index [19], and the Hospital Anxiety and Depression Scale [20,21])

Medication (0,3,6,9,12,15 Months)	Medication

Legend: AS patients in the PAS cohort were asked to consent to completing questionnaires either online if they have internet access, or by post. A website has been developed to give access to the questionnaires http://www.ashealth.co.uk/

The use of HERALD methodology can stratify groups of patients to identify the early characteristics of patients who subsequently develop severe disease, thus, enabling these patients to be targeted with early aggressive therapy in order to prevent severe damage and need for surgery. This profiling can be used to estimate the potential resource savings of focusing treatment on those patients with patterns of disease suggestive of the development of a severe outcome. All these will directly affect patient care for AS in terms of informing NHS service provision and NICE guidelines for the use of expensive biological therapies, and informing the process of assessment of cost-effectiveness. In principle, the methods developed for the PAS cohort and described here can be extrapolated to be used in other chronic disease conditions [6]; thus improving patient care for all those conditions. Linked routine data provides many opportunities for enhanced healthcare research and allows evaluation of impacts beyond the limited primary outcomes of interventional studies. As an example, the expanding SAIL databank in Wales already holds over a billion anonymised records from various databases, which can be anonymously linked at the individual record level. The combination of routine data with information from patients and RCTs allows the validation of real-life data and its application for clinical research. These linkable databases provide factual and continuous information with rich clinical and non-clinical details, which offers wide ranging opportunities in the realm of conducting evaluative research, clinical epidemiology, trial recruitment, genetic research, basic research of biological markers, stratified medicine, post-trial surveillance, risk assessment, service delivery evaluation, resource use, decision analysis, identification of early disease predictors, and the identification of subjects for prospective studies [6,22,23]. This data system also offers the opportunity for post-marketing surveillance and pharmacovigilance of new expensive, and often potentially dangerous, healthcare interventions in real-life settings. Complementing this resource with targeted health economic analysis, as proposed in the HERALD methodology, offers a unique opportunity to deliver the level of health economic data required to evaluate and drive forward cost-effective modern healthcare services.

## Conclusion

The linkage of routine data, patient completed questionnaires and trial data offers unique opportunities for enhanced health economic analysis, including assessment of the validity, reliability and generalisability of health economic data not possible through the use of traditional isolated datasets. The information obtained from the linked data system would help improving patient pathways, and thus maximise clinical efficiency and efficiency in the use of resources.

## Competing interests

The authors declare that they have no competing interests.

## Authors' contributions

All authors were involved in the design of the HERALD protocol. The first draft of the paper was written by MJH, SB, and SS. Subsequent drafts were amended and finally approved by all the authors.

## Authors' information

MJH is a Lecturer in Economics at Keele Management School, Keele University; and also worked as a health economist at the College of Medicine, Swansea University during 2010/11. SB is a Reader in Epidemiology and has worked for 15 years in the field of spondyloarthropathy. SS is a Clinical Senior Lecturer and Honorary Consultant Rheumatologist (MBBCh, MRCP, PhD) whose specialist clinical and research interest is Spondyloarthritis.

## Pre-publication history

The pre-publication history for this paper can be accessed here:

http://www.biomedcentral.com/1472-6947/12/24/prepub
